# DichroIDP: a method for analyses of intrinsically disordered proteins using circular dichroism spectroscopy

**DOI:** 10.1038/s42003-023-05178-2

**Published:** 2023-08-08

**Authors:** Andrew J. Miles, Elliot D. Drew, B. A. Wallace

**Affiliations:** 1 Institute of Structural and Molecular Biology, Birkbeck University of London, London, WC1E 7HX UK; 2 School of Biological and Chemical Sciences, Queen Mary University of London, Mile End Road, London, E1 4NS UK; 3 Present Address: Zappi, London, NW1 7JN UK

**Keywords:** Computational biology and bioinformatics, Protein databases, Computational biophysics

## Abstract

Intrinsically disordered proteins (IDPs) are comprised of significant numbers of residues that form neither helix, sheet, nor any other canonical type of secondary structure. They play important roles in a broad range of biological processes, such as molecular recognition and signalling, largely due to their chameleon-like ability to change structure from unordered when free in solution to ordered when bound to partner molecules. Circular dichroism (CD) spectroscopy is a widely-used method for characterising protein secondary structures, but analyses of IDPs using CD spectroscopy have suffered because the methods and reference datasets used for the empirical determination of secondary structures do not contain adequate representations of unordered structures. This work describes the creation, validation and testing of a standalone Windows-based application, DichroIDP, and a new reference dataset, IDP175, which is suitable for analyses of proteins containing significant amounts of disordered structure. DichroIDP enables secondary structure determinations of IDPs and proteins containing intrinsically disordered regions.

## Abstract (web summary)

DichroIDP is a method with a bespoke reference dataset for analyzing and determining secondary structures in proteins containing intrinsically disordered regions using circular dichroism spectroscopy.

## Introduction

Most globular proteins in their native state are primarily comprised of canonical (helical, sheet and turn) secondary structures and exist in well-defined conformations with specific three-dimensional structures. In contrast, intrinsically disordered proteins (IDPs) tend to form dynamic ensembles of highly flexible polypeptide chains that often have very limited amounts of persistent secondary structures^1^. In addition, some globular proteins also exhibit intrinsically disordered regions (IDRs) comprised of ~30 or more consecutive amino acid residues, which do not adopt regular secondary structures^2^. Due to their flexible nature, IDPs and proteins with IDRs have the potential to bind to a range of partner molecules, acquiring different conformations according to the templates provided by the binding partners. This is likely to be a reason why they appear to be involved in a number of regulatory functions, including molecular recognition and signalling^3^. In humans, for instance, ~80% of “hub” proteins with >10 known binding partners are predicted to contain long disordered regions^4^.

Circular dichroism (CD) spectroscopy (and the related method of synchrotron radiation circular dichroism (SRCD) spectroscopy^5^) are widely-used techniques for quantitatively analysing the helix, sheet and turn contents of proteins^6,7^ in different environments and as components of complexes. In most cases, the analyses employ empirical methods that rely on the availability of suitable and broadly-based reference datasets (RDS) derived from proteins with known crystal structures^8–10^. These types of analyses, however, can be of limited value if the protein to be analysed includes significant numbers of residues that are not present in canonical types of secondary structures. Such residues usually have been grouped together under nomenclatures such as “other”, “unordered”, “irregular”, “disordered” or “random coil”. Empirical analyses of proteins with such features rely on the availability of examples of protein spectra which include non-canonical structures in their reference datasets; however currently-available reference datasets used by the CD methods have been derived from proteins that crystallise, and therefore tend to include only limited numbers of examples of natively “unordered” or disordered types of secondary structure (which tend to be missing in crystal structures) and are often referred to as “other”. Indeed, computationally, the “other” type of secondary structure is often simply ascribed to the remainder of the protein that is not calculated to be helical, sheet, or in some cases, turn. As examples, “other” type structures have also been used to refer to loop structures that do not form the strict hydrogen-bond pairings present in different types of tight turns, to unfolded structures present in thermally- or chemically- treated proteins which have lost their tertiary structural interactions, or to intrinsically disordered regions (IDRs) of proteins which do not adopt regular helical or sheet structures.

The aim of this study was therefore to improve the coverage of disordered secondary structure types available in CD reference databases, and the methods used for their analyses by CD spectroscopy. It describes a new reference dataset which includes examples of this class of structure, and an associated novel application method that can be used to analyse CD spectra from a wide-range of protein types, including IDP and IDR-containing proteins.

A number of existing secondary structure analysis tools^11–13^ have been developed which incorporate or are based on different empirical algorithms for determining the secondary structures of proteins from CD spectroscopic data using the available reference datasets derived from spectra of globular proteins with known crystal structures. These tools include the SELCON3^14^, CONTINLL^15^, CDSSTR^16^, and BeStSel^12^ deconvolution algorithms, and the SESCA^13^ programme, amongst others. Although there is usually some variation in the results obtained with these different algorithms, the majority of the differences arise not from the different methodologies, but rather from the use of different reference datasets comprised of different proteins^11^. To date, the available reference datasets with the most comprehensive coverage of protein secondary structure and fold space are the bioinformatics-designed SP175 reference dataset^8^ (for soluble proteins), the SMP180 reference dataset^9^ which includes both soluble and membrane proteins, and the SP175+ reference dataset (SP175 augmented by a number of additional beta sheet proteins^17^). The first two of these are included with the DichroWeb analysis server^7,11^ and the latter is available in the BeStSel^12^ analysis server. The SESCA^13^ programme utilises a number of datasets, including a modified version of SP175.

However, none of the currently available reference datasets contain representatives of proteins that include significant amounts of intrinsic disorder. This is primarily because the disordered regions in globular proteins tend not to be visible in crystal structures, and because IDPs, by their nature, do not form regular crystallisable structures, even though they may contain regions that are statically- or dynamically- well-defined. One existing reference dataset, CDPro42^10^ from the CDPro software package (dataset 7 available in the DichroWeb^11^ online analysis resource located at: http://dichroweb.cryst.bbk.ac.uk/html/home.shtml), does contain several spectra of denatured proteins as representatives of “disordered” proteins, which are assumed to be comprised of ~90% unordered structure; but there is no independent evidence that they adopt such structures nor that these denatured structures (produced by chemical unfolding reagents or heating) are related to intrinsically-unfolded regions of native proteins. In order to create a new reference dataset (and an associated analysis method) that distinguishes disordered structures from helix, sheet and turn, it has been necessary to include examples of IDP proteins (Supplementary Table S1 (top)) with those of standard globular proteins that are primarily composed of canonical helical and sheet secondary structures (Supplementary Table S1 (bottom)). However, since IDPs do not readily crystallise, several new bioinformatics methods have been used to predict secondary structures for a number of IDP or IDR-rich proteins directly from their primary sequences. These methods include Spot-1D^18^, NetSurfP-2.0^19^, RaptorX^20^ and AlphaFold2^21^. All predict solvent accessibility and backbone dihedral angles, and therefore the potential secondary structure of individual residues in the sequence, using deep learning neural networks trained on structures present in the Protein Data Bank (PDB)^22^ (Supplementary Table S2). Spot-1D and NetSurfP-2.0 output three- and eight-state residue-by-residue secondary structure predictions, whereas RaptorX and AlphaFold2 output atomic coordinates in PDB format. The latter two methods permit secondary structures to be independently calculated using the dictionary of protein secondary structure (DSSP)^23^ algorithm (in the same way as those used for the soluble proteins in the dataset, which all have crystal structures available in the PDB).

The use of the AlphaFold method to predict protein structures in general has been endorsed by the Critical Assessment of Protein Structure Prediction (CASP)^24^ assessment competition, which compared leading structure prediction methods in detail for a wide range of proteins. AlphaFold was the top-ranked method overall, with a median GDT (Global Distance Test) score of 92.4 across all targets and 87.0 on the challenging free-modelling category, compared to 72.8 and 61.0 for the next best methods in these categories. However, those assessments were done primarily on fully-ordered proteins, rather than the disordered or partially disordered proteins in the present study. IDPs (or ordered proteins with IDRs) are different types of structures than fully ordered proteins, however, David et al.^25^, Ruff and Pappu^26^ and Wilson et al.^27^ have asserted, that whilst the details of the AlphaFold2 predictions of the 3D structures of the IDP regions may not be exactly defined residue-by-residue, what is clear is that the extent and characteristics of the IDP region residues are clearly indicated by Alphafold2 to be IDRs in nature. This is the information required for the present study.

**Fig. 1:**
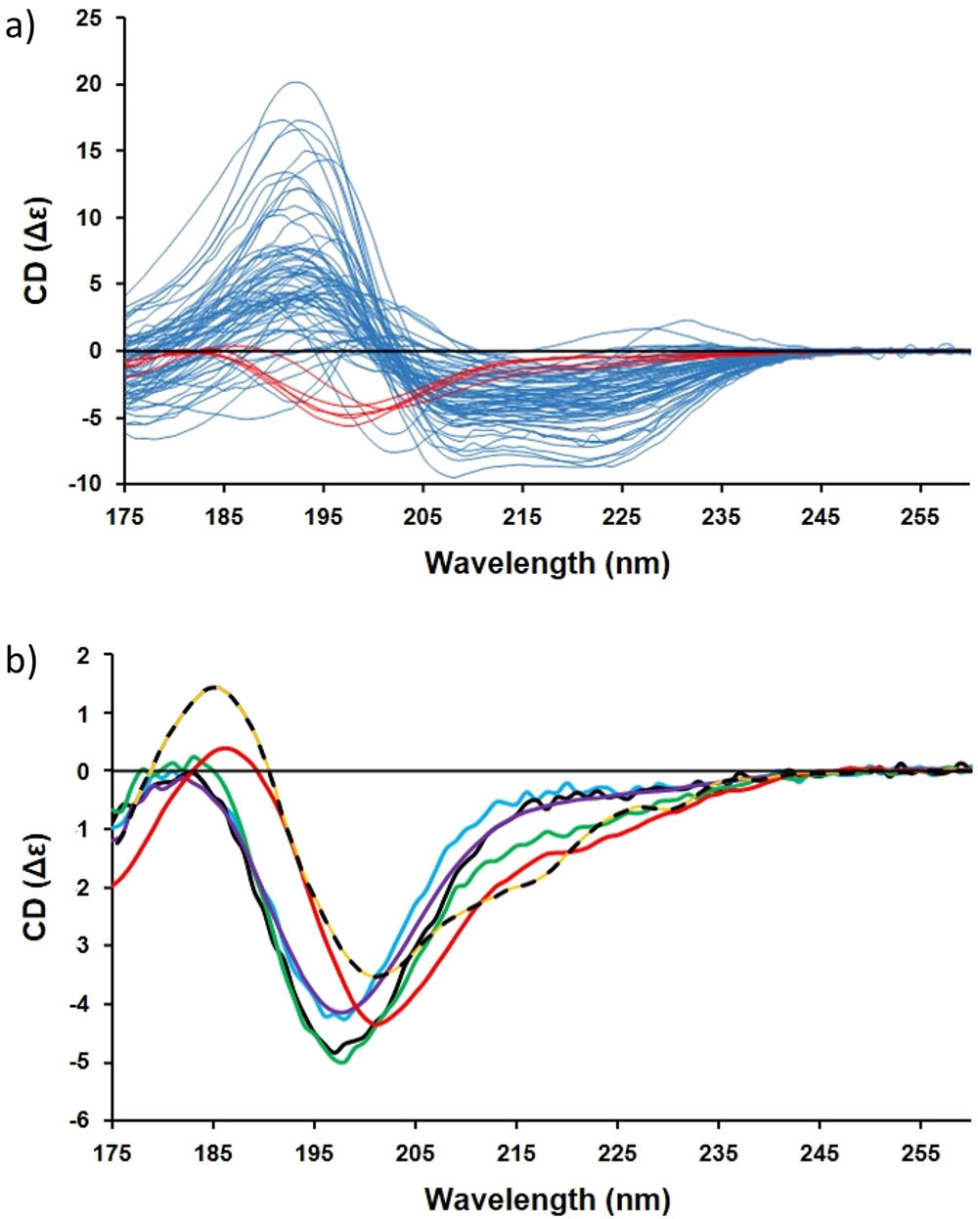
CD spectra associated with the IDP175 reference dataset. In (**a**), the complete IDP175 dataset where the new IDP spectra (listed in Supplementary Table S1 (top)) are shown in red whilst the spectra of the SP175^8^ proteins (listed in Supplementary Table S2) are shown in pale blue. The new IDP spectra of the IDP175 dataset in (**b**) are of MEG-14^33^ (purple), HASPA^30^ (blue), HASPB^30^ (black), TARP^28^ (green), and casein^28^ (red). For comparison, the dotted line is the spectrum of alpha-chymotrypsinogen (not included in the IDP reference dataset), which contains a highly-twisted right-handed beta-sheet, illustrating the similarity of this type of spectrum (designated “β2”) to the spectra of the IDPs.

The new reference dataset reported herein is designated IDP175, a name which reflects the inclusion of intrinsically disordered proteins with the low wavelength end of their spectra extending down to a wavelength minimum of 175 nm. It includes spectra (Fig. 1) from both the existing SP175 RDS^8^ and the newly-characterised group of IDP protein spectra determined in this study but which are not present in any other dataset available to date. All components are publicly-available in the Protein Circular Dichroism Data Bank (PCDDB)^28^. This new dataset should therefore be appropriate for analyses of not only IDPs, but also for proteins which contain mixtures of both ordered and disordered structures. For ease of use, the IDP175 dataset has been incorporated into a stand-alone Windows application method called DichroIDP, which utilises SelMat^8^ a modified version of the SELCON3 algorithm to determine secondary structures from protein CD spectra.

The IDP175 reference dataset was first cross-validated by leave-one-out procedures using a modified version of DichroIDP that was produced exclusively for the purpose of testing. The IDP175 reference dataset was then trialled in the DichroIDP app using spectra of both IDPs and spectra of globular proteins with significant amounts of disorder, in order to demonstrate its general suitability; the results obtained were compared with results using three existing RDS, SP175^8^, CDPro^10^ and SP175+ ^17^ In the cross-validation tests, the IDP175 and other reference datasets produced roughly comparable results for helix and sheet components, but the IDP175 reference dataset produced a significant improvement for the calculated turn and disordered components based on the Pearsons correlation and zeta factor criteria^8^. More crucially, whilst producing similar values for helix and sheet components, IDP175 outperformed all other reference datasets, and also other widely used methods, including BeStSel^12^ and K2D3^29^, in analyses of the spectra of disordered proteins, defined by how close the values were to those calculated by the DSSP algorithm based on either their AlphaFold2^21^ or PDB^22^ structures.

## Results

### Criteria used for selection of intrinsically disordered proteins

The new proteins in the IDP175 RDS (Supplementary Table S1 (top)) ranged from small (49 residues, the region 174-222 of the translocated actin-recruiting phosphoprotein (Tarp_174-222_) from *Chlamydia trachomatis*) to moderate size (>300 residues, the hydrophilic acylated surface protein from *Leishmania major* (HASPA))^30^. Only soluble (not membrane) proteins were included, and no proteins with bound chromophores or ligands that absorb in the UV or visible ranges were included, as these could potentially distort the protein spectra, even in the far UV region used for secondary structure analyses.

### Choice of proteins included in either the reference or test datasets

The difficulty in expressing and purifying soluble monomeric IDPs meant that there were a limited number of fully IDP proteins or polypeptides available for use. Furthermore, as pointed out by Micsonai et al.^31^, bioinformatics methods for obtaining secondary structure from protein sequences do not take into account environmental factors, which can radically alter a protein’s conformation. Therefore the number of IDP spectra available was further limited as some of the structural data obtained using these methods were deemed to not match the general form of the CD spectra obtained. Consequently judicious choices had to be made regarding which of the IDP proteins were to be used for creation of the RDS and which were to be used for testing of the RDS. The more proteins in the reference dataset, the more accurate it was likely to be; however, including more of the proteins in the RDS would then limit the number of test proteins available for independent validation calculations. Ultimately the selection of proteins included in the RDS was guided by optimisation of the cross-validation test parameters (see below). The proteins that have been included in IDP175 and those used for testing are listed in Supplementary Tables S1 (top) and S1 (bottom). The spectra of the components of the entire dataset and of only the “fully IDP” proteins included in the dataset are shown in Fig. 1a, b, respectively; not surprisingly, all of the IDP spectra appear to be very similar.

The spectra of alpha-chymotrypsin, alpha-chymotrypsinogen, elastase and soybean trypsin inhibitor, which contain right-hand-twisted beta-sheets (hereafter designated β2 spectra) can resemble the spectra of the IDPs^12,31^, (see Fig. 1b), causing existing analysis algorithms to assign excessive beta structure to IDP spectra if they are included in the RDS. This was also found to be the case for the IDP175 dataset, especially if there are (even very small) errors in the spectral magnitudes due to inaccurate concentration determinations or cell pathlength measurements. β2 spectra were therefore removed from the IDP175 RDS, along with the spectrum of ferredoxin which gives an anomalous disordered-like spectrum, likely due to the presence of its chromophore.

**Fig. 2:**
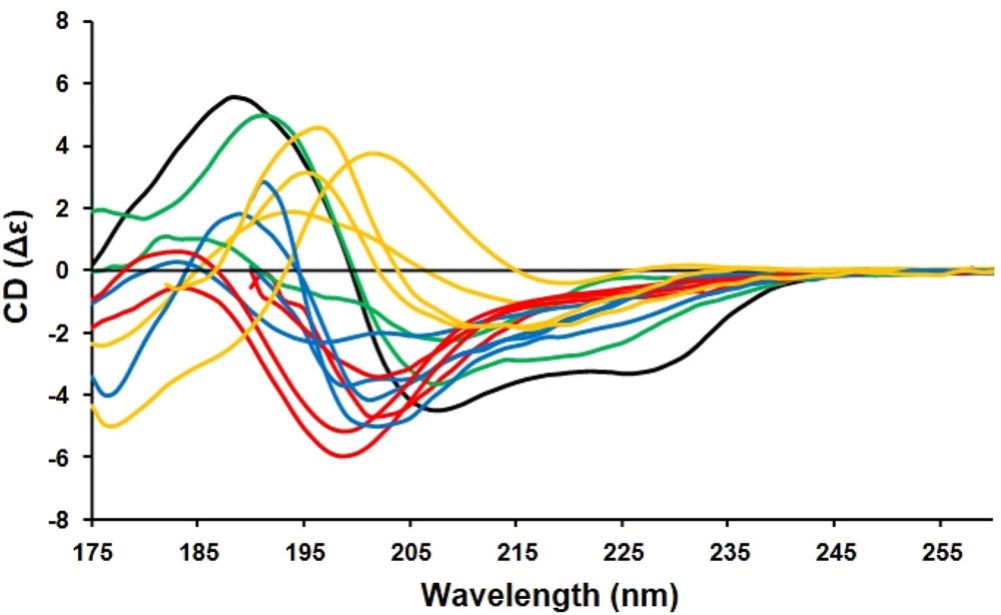
CD spectra of the test proteins (see details in Supplementary Table S1 (bottom)). These include the following types of proteins: intrinsically disordered proteins (in red) osteopontin, amelogenin, Sic1, BB1 C-terminus; Beta-1 proteins (in yellow) β2-microglobulin, prealbumin, Bence Jones Protein, eGFP; Βeta-2 proteins (in blue) MAGI-1 PDZ1, UTPase, trypsin, ecotin; and mixed αβ proteins (in green) pokeweed lectin, and saporin; and the primarily alpha helical protein (in black) α-lactalbumin. Details of their secondary structures and their PCDDBIDs are listed in Supplementary Table S1 (bottom).

The test dataset included not only IDPs, but folded proteins with mostly beta sheet (Types 1 and 2 (relaxed and right-hand-twisted)) structures, proteins comprised of both alpha helix and beta sheet, and alpha helical proteins (Fig. 2). The latter inclusions were to demonstrate how the RDS performed in analyses of all common secondary structure types.

### Protein spectra sources and selection

Secondary structures of the SP175^8^ proteins in the dataset were derived from crystal structure coordinates (from the same PDB files that were used for the original SP175 dataset) using the DSSP^23^ algorithm (Supplementary Table S2). The Spot-1D^18^, NetsurfP-2.0^19^, RaptorX^20^, and AlphaFold2^21^ prediction methods were used to generate structural data from the primary sequences of the IDP proteins, which do not readily crystallise and therefore had no crystal structures included in the PDB^22^. Although the results were similar, RaptorX^20^ and AlphaFold2^21^ were initially favoured in this study because they generate PDB files that can be analysed in the same manner as globular protein structures, using DSSP^23^ and AlphaFold2 (which was endorsed by CASP^24^ results). Of all four methods, AlphaFold2^21^ was judged to give the best performance in cross-validation results and in the analyses of IDPs in the test dataset, with respect to the disordered fraction (Supplementary Table S3). Therefore only structures obtained from this method were used in the final RDS. Four protein spectra for which structural assignments did not correlate with the appearances of their CD spectra, were discarded (Supplementary Table S4). Although Wilson et al.^27^ suggested that the problem usually manifests in the over-prediction of disordered residues, the discarded proteins were predicted to have more alpha helix by AlphaFold2 than was judged to be the case from the general appearance of the CD spectra (Supplementary Fig. S1). For example, the CD spectrum of alpha synuclein in water indicates a disordered structure with a single negative peak at around 200 nm. However AlphaFold2 assigns 45% helix to this protein, a structure which would generate a spectrum with noticeable negative peaks at ~222 nm, ~208 nm and a positive peak ~190 nm.

### Definitions of secondary structural classifications used in IDP175

When defining the number of separate classes to be identified from CD spectroscopic data, it is important to consider the information content present in the spectral data: if the data extend down to 190 nm, they have high enough information content^8^ to distinguish only 5 different types of secondary structures, although this number increases to 7 or 8 if data down to 175 nm (which can be achieved using SRCD instruments) is included. The secondary structural components of two of the most popular general datasets, SP175^8^ and SMP180^9^, use the six structural classifications of regular helix, distorted helix, regular sheet, distorted sheet, turns and “other”, where “other” combines everything else. The SP175+ dataset^17^, used in the BeStSel server^12^ (which was primarily designed to analyse beta sheets) divides the components into helix, parallel and antiparallel beta sheet, turns and other. However, in the present study, since we are mainly concerned with accurate predictions of the ‘other’ component, and to prevent over interpretation when data only reaches 190 nm, our output was limited to four categories. These are based on their DSSP values where the DSSP classes H, G and I are combined as helix, sheet is class E, turn is a combination of classes T and S and disorder is everything else (classes B and O).

### Validations and dataset analysis comparisons

Cross-validation studies were first done for all four standard secondary structure types (helix, sheet, turn and disordered) using the “leave one out” method^8^ (Table 1) in order to show that there is adequate coverage of representative types present in the new IDP175 reference dataset and in a version of the dataset with the low wavelength cutoff of the data truncated to 190 nm (designated IDP175t). The selection of proteins to be part of the RDS (as opposed to test proteins) was optimised to produce the highest correlations for all four categories.

**Table 1:** Comparison of the leave-one-out cross-validations using the DichroIDP method with the dictionary of secondary structure of proteins (DSSP)^23^ assignment method.

**a) IDP175**				**b) IDP175t**			
	r	δ	ζ		r	δ	**ζ**
H	0.9270	0.0801	2.6633	H	0.9214	0.0831	2.5668
E	0.8543	0.0886	1.9186	E	0.8422	0.0920	1.8476
T	0.5342	0.0613	1.1365	T	0.5411	0.0599	1.1631
D	0.9322	0.0649	2.6987	D	0.9364	0.0617	2.8400
**c) SP175**				**d) SP175t**			
	r	δ	ζ		r	δ	**ζ**
H	0.9299	0.0771	2.7191	H	0.9233	0.0807	2.5977
E	0.8398	0.0871	1.8354	E	0.8018	0.0956	1.6715
T	0.3691	0.0543	1.0353	T	0.3915	0.0535	1.0510
D	0.5945	0.0535	1.3051	D	0.7282	0.0484	1.4414
**e) CDPro42**				**f)**	**SP175+**^**17**^ **(from BeStSel**^**12**^**)**		
	r	δ	ζ		r	δ	ζ
H	0.9175	0.0870	2.5001	H	0.9087	0.0897	2.3924
E	0.6980	0.1145	1.3710	E	0.8016	0.1042	1.6712
T	0.5296	0.0771	1.0873	T	0.4454	0.0531	1.0967
D	0.7385	0.1550	1.3511	D	0.6623	0.054	1.3077

The following reference datasets were used: a) IDP175; b) IDP175t (low wavelength cut off 190 nm); c) SP175^8^, d) SP175t (low wavelength cut off 190 nm); e) CDPro42^10^ and f) SP175+^17^. The statistical parameters reported are: r, the Pearson’s correlation coefficient; δ, the root mean squared deviation, and ζ the ratio of δ over the population standard deviation as defined in the main text. The cross-validation values for all of the reference datasets/assignment methods are similar for helix and sheet secondary structures, but the disordered structure contents are very much improved using the IDP175 and IDP175t reference datasets. H,E,T, and D refer to the helical, sheet, turn, and disordered components, respectively.

The cross validation results (Table 1) were compared with studies using the SP175^8^ and SP175t RDS (like IDP175t, SP175t uses data to 190 nm, as opposed to the SP175 reference dataset which requires data to 175 nm), CDPro^10^ (cutoff 190 nm) and SP175+ ^17^ using the same 4 secondary structural types defined in IDP175, so that the quality of the analyses could be directly compared. All datasets exhibited little difference in the quality of the analyses of the helix and sheet categories, but IDP175 and IDP175t showed significant improvements for the turn and “disordered” categories, as expected.

**Fig. 3:**
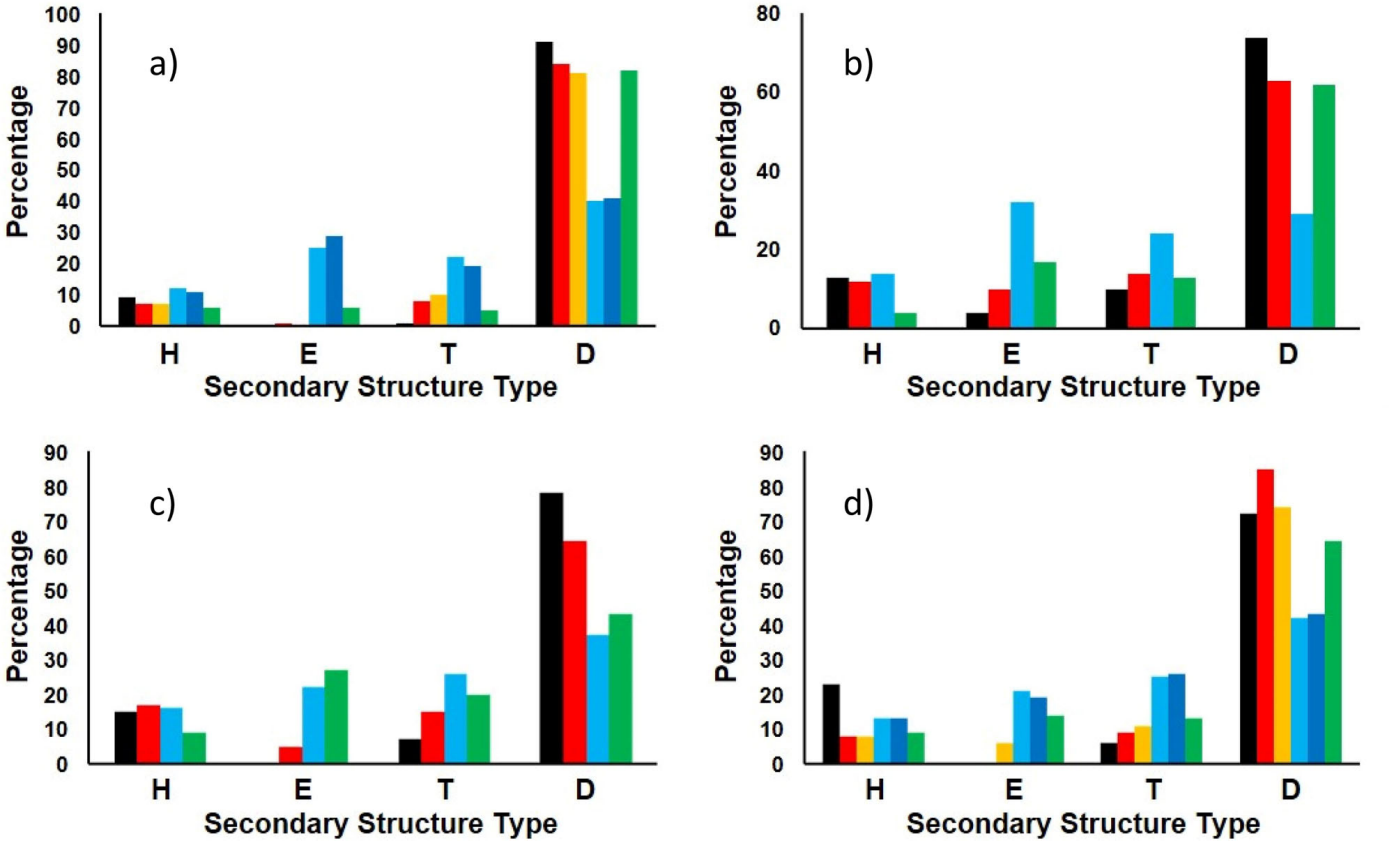
Secondary structure analyses of the IDPs in the test set using DichroIDP in conjunction with various reference datasets mentioned in the text compared with AlphaFold2 predictions. The proteins are: (**a**) osteopontin; (**b**) Sic1^36^; (**c**) amelogenin^37^, and (**d**) ΒB1 C-terminus^35^. Secondary structures are indicated as: H=Helix (using “Dictionary of Secondary Structure of Proteins” (DSSP)^23^ assignments H + G + I). E= beta strand (DSSP E). T= Turn (DSSP T + S). D=disordered (DSSP B + O). For each secondary structure type, the coloured bars show the % of each structure predicted from their CD spectra using the following datasets: (red) IDP175t, (yellow) IDP175, (light blue) SP175t, (dark blue) SP175^8^, (green) CDPro42^10^. These are compared to AlphaFold2^21^ predictions depicted in black in each panel. Sic1 and amelogenin were only analysed using the datasets IDP175t, SP175t and CDPro42 because their data had low wavelength cutoffs above 175 nm. Protein details are listed in Supplementary Table S1 (bottom).

Then de novo tests were done using the spectra of IDPs and folded proteins (shown in Fig. 2b) not present in the reference dataset. The results with IDP175 or IDP175t (depending on the low wavelength cutoff of the test protein data) are compared once more with the other reference datasets mentioned above (Fig. 3 and Supplementary Table S5) and also with results obtained using BestSel^12^ and K2D3^29^, a neural network method trained on spectra predicted from PDB structures using DichroCalc^32^ (Supplementary Table S6). The disordered test proteins were also analysed using SESCA^13^ with the IDP175 dataset and DSSP-F, a dataset that comes with the SESCA package (Supplementary Table S7). The calculated secondary structure contents using the IDP175 reference dataset produced values that were closer to those of AlphaFold2 than those produced by the DSSP-F reference data. In addition, the NRMSD values were generally smaller.

## Discussion

Early CD spectroscopic secondary structure analysis methods divided proteins into helical, sheet or “random coil” types of secondary structures, and used very limited numbers of proteins with known structures to create reference datasets for simple deconvolutions. Later methods used selection methods^14–16^, generally with reference datasets consisting of slightly larger numbers of proteins containing representative types of secondary structure. Their secondary structural components were, for the most part, divided into regular helix, distorted helix, regular sheet, distorted sheet, (sometimes) turns and “other” structures, where “other” combines everything else, including residues present in undefined regions of the protein crystal structure. More recently CD reference datasets for soluble^8^ and membrane proteins^9^ have been developed using bioinformatics techniques enabling wider coverage of fold and secondary structure spaces.

Most reference datasets available to date include only proteins whose crystal structures are known^8,9^, but at least one reference dataset^10^ included a few “denatured” protein structures (produced by acid and heat denaturation) all of which were assumed to contain 90% disordered structure. The availability of a number of stable purified, soluble IDPs, has now enabled the measurements of their CD spectra whilst the emergence of deep learning neural networks such as AlphaFold2^21^, which has been shown to outperform other prediction methods in the Critical Assessment of Protein Structure Prediction exercises, now means there is a method for assigning atomic coordinates to this additional class of protein, which had not proved to be amenable to crystallisation. Both of these developments have thus allowed the construction, validation and testing of a new reference dataset for use with the new DichroIDP application described herein, to characterise proteins that have considerable amounts of disordered structure, often in the presence of canonical secondary structures.

In summary, we have produced a new user-friendly tool for studying an important class of proteins which are disordered or partially disordered, enabling quantitation of the amount of disordered structure present in both primarily folded, and primarily unfolded proteins using CD data. Previously, this class of proteins was not accurately analysed by CD due to the methodologies available and the lack of suitable reference and test protein spectra.

## Materials and methods

### Materials

The IDP175 reference dataset included the following spectra obtained previously in our lab: MEG-14 (microexon 14 protein from *Schistosoma mansoni*)^33^, HASPA and HASPB (hydrophilic acylated surface proteins from *Leishmania* major)^30^, bovine casein (Sigma-Aldrich), β-b1 C-terminus^34,35^, and TARP_174-222_ (translocated actin recruiting phosphoprotein from *Chlamydia trachomatis*, donated by Prof. Tharin Blumenschein of the University of East Anglia). Test proteins included four soluble proteins: Bence-Jones lambda protein, bovine trypsin, prealbumin and alpha-lactalbumin present in the SMP180 RDS^9^ (which were not in the SP175 RDS^8^), plus pokeweed lectin and saporin (Sigma-Aldrich). The spectra of six additional proteins (osteopontin, UTPase, ecotin, β2-microglobulin, MAGI-1PDZ1 and eGFP) were obtained from existing PCDDB^28^ entries. Two other IDP test protein spectra were obtained by digitising published spectra of cyclin-dependent-kinase inhibitor, Sic1^36^, and strepsirrhine primate amelogenin^37^ using the desktop version of WebPlotDigitiser^38^. The CD spectra of all of the IDP175 proteins are depicted in Fig. 1 (main text), whilst the CD spectra of the test proteins are in Fig. 2 (main text). The secondary structures and UniProt^39^ codes of all of these proteins (and, where available, their PCDDB IDs) are listed in Supplementary Tables S1 (top), S1 (bottom), and S2.

## Methods

### Synchrotron radiation circular dichroism spectroscopy

All synchrotron radiation circular dichroism (SRCD) spectra that have not been previously published were measured at synchrotron beamlines CD1 or UV1 at the ISA facility in Aarhus, Denmark except for β-b1 C-terminus, which was measured on beamline CD12 at the SRS, Daresbury, UK.

The protein concentrations were determined by the A_280_ method with extinction coefficients calculated using the EXPASY webserver^40^. For comparison, the concentrations of proteins measured on beamlines CD1 and UV1 were also determined in situ using the A_205_ method whereby the sample absorbance is determined from the HT (high tension) signal and the synchrotron ring current^41^, and the concentration determined using amino acid extinction coefficients at 205 nm from values by Anthis and Clore^42^.

Spectra were obtained at 20°C in quartz cylindrical demountable cells (Hellma UK, Ltd) with optical pathlengths of 0.0015, 0.0024, or 0.0011 cm (each calibrated using the interference method^43^). In all cases the dataset spectra were measured from a high wavelength of >260 nm down to a low wavelength of a least 175 nm, in 1 nm steps, using averaging times of 1 to 3 s. Data processing was carried out using the CDtoolX software^44^ as follows: Three replicate sample spectra were averaged and a buffer baseline (also the average of three replicate spectra) subtracted. The net spectrum was calibrated using a spectrum of camphorsulphonic acid^45^ measured on the same instrument and then scaled to delta epsilon units.

The protein spectra were divided into those incorporated into the reference dataset (Supplementary Table S1(top)) and the test dataset (Supplementary Table S1 (bottom)), following cross-validation testing (see below) to optimise the reference dataset contents whilst retaining availability of some of the other spectra for validation testing. The new RDS spectra were added to 66 spectra from the SP175 RDS^8^ obtained from the PCDDB^28^. In addition, a number of the SP175^8^ entries have been updated in the PCDDB^28^ and indicated by ‘1’ in the 10th position of the PCDDBid.

### Methods for assignment of secondary structures

The AlphaFold2^21^ website (https://colab.research.google.com/github/sokrypton/ColabFold/blob/main/AlphaFold22.ipynb#scrollTo=kOblAo-xetgx) was used to produce structures from the IDP protein sequences with the default settings that produce five models for each sequence. The helix, sheet, and turn secondary structure percentages of the proteins were defined by their DSSP^23^ classifications using the 2Struc^46^ webserver. Residues defined as H (alpha helix), G (3_10_ helix) and I (pi helix) were combined and classified as helix, and the DSSP E class was assigned as beta strand. DSSP S (beta loop) and T (bonded turn) classes were designated “turn”, whereas B (bend) was combined with the remainder and designated disordered. The values obtained from the five top AlphaFold2^21^ models for each IDP protein were averaged. AlphaFold2 models were also produced for the folded test proteins for comparison. DSSP^23^ values for the SP175^8^ proteins and folded test proteins were calculated from their crystal structures in the PDB^22^ (where available) using the 2Struc webserver^46^ (Supplementary Tables S1 (top) and S1 (bottom)).

### Method for CD-based calculations of secondary structure

The IDP175 reference dataset was incorporated into the selectable list of available reference datasets in the DichroIDP standalone application produced using the Qt framework^47^. It uses the existing SelMat^8^ algorithm, rewritten in C + + using the ALGLIB^48^ package and can be used for analysing spectra that contain data between a high wavelength of at least 240 nm and any low wavelength between 200 and 175 nm. SelMat^8^ is a version of SELCON3^14^ where the sum, fraction and helix rules are relaxed to give at least one solution for any protein spectrum, and was originally written for MATLAB^49^. Spectra can be scaled if necessary before analysis. The output consists of a table showing results from all stages of the algorithm calculation and includes a list of the closest proteins in the dataset to the query spectrum. The final result is presented in a second table that includes the normalised root mean square deviation (NRMSD^11^) between the query data and the back-calculated spectrum (which is displayed along with the query spectrum for comparison). The RMSD is normalised because it does not take into account the relative magnitude of the spectral fitting error. For example, where the CD signal is small in magnitude, error bars will exaggerate the error compared to where it is large in magnitude. The widely-used “NRMSD” parameter attempts to rectify this, and is defined as:

1 $${NRMSD}=\sqrt{\frac{{\sum }_{\lambda }{\left({\theta }_{\exp }-{\theta }_{{calc}}\right)}^{2}}{\mathop{\sum}\limits_{\lambda }{({\theta }_{{calc}})}^{2}}}$$

where *θ*_*exp*_ and *θ*_*calc*_ are the experimental and back-calculated ellipticities, respectively, at each data point in the spectrum, with lower values indicating a closer match between experimental and reference data. The NRMSD (calculated in the same way for all methods) depends on how close the query spectrum is to an average of the nearest selected spectra in the dataset, from which the back-calculated spectrum is calculated. This means that it does not always reflect the accuracy of the secondary structure estimate in every case. This is demonstrated when for example a disordered spectrum is analysed using SP175 (or the BeStSel reference database), or when analysing a β2 spectrum using IDP175. However, the NRMSD does usually give a good indication of accuracy when using an appropriate dataset for analysing the query protein. Hence we have created a number of datasets over the years for different types of proteins, including the IDP dataset reported in this study. The result tables (or any part of them) produced by DichroIDP can be pasted directly into spreadsheet software. There is an extensive help file associated with the app which can be accessed directly from its “help” menu.

### Validation and testing

The IDP175 and IDP175t reference datasets were cross-validated using the leave-one-out approach in a modified version of DichroIDP. Statistical parameters are the Pearsons correlation coefficient (*r*) and the root mean square deviation (δ). The zeta (ζ) value, which is the ratio of δ over the population standard deviation is defined (as previously reported^8^) as follows:

2 $${{{{{{{\rm{\zeta }}}}}}}}=\frac{{{{{{{{\rm{\delta }}}}}}}}}{{\sigma }_{x}}$$

where σ_X_ is the standard deviation of the calculated fractions of secondary structure x. Values of ζ = < 1 indicate a value no better than a guess whereas values of 2-3 are statistically significant. Higher values of r and lower values of δ correspond to better cross-validation performances. The results (Table 1) were compared with the cross-validation of the SP175^8^, SP175t, CDPro^10^, and SP175+^17^ reference datasets using the new definitions of secondary structure classes.

The reference datasets were then tested for accuracy with the IDP test dataset of spectra of related and unrelated IDPs (Fig. 3 and Supplementary Table S1 [bottom]). Other proteins with [alpha + beta] contents of ≤40%, and thus significant amounts of “other” structure based on their crystal structures, were also included in the test dataset. The test results were compared to those obtained using datasets SP175^8^ CDPro^10^ and SP175 + ^17^ with DichroIDP and the secondary structure assignments mentioned above (Supplementary Table S5). Further comparisons were made using the results from the BeStSel^12^ and K2D3^29^ servers (Supplementary Table S6) using the secondary structure assignments discussed in references ^12^ and ^25^ respectively, and also using the SESCA^13^ method in conjunction with the IDP175 and DSSP-F RDS (Supplementary Table S7) with the secondary structure assignments used in DichroIDP.

### Reporting summary

Further information on research design is available in the Nature Portfolio Reporting Summary linked to this article.

### Supplementary information

Supplementary Information

Reporting Summary

## Data Availability

The new reference dataset spectra described in this paper, and their associated metadata, have been deposited in the Protein Circular Dichroism Data Bank (PCDDB)^28^ (located at http://pcddb.cryst.bbk.ac.uk). They include the following proteins: HASPA, HASPB, casein, and Tarp_174-222,_ with consecutive records CD0006406000 to CD0006409000; they are identified by the keyword “IDP175”. MEG-14^33^ was already present in the PCDDB with PCDDBid CD0004064000. The test protein spectra have also been deposited in the PCDDB with the following PCDDBids: osteopontin, CD0003667000; β2-microglobulin, CD0003894000; Bence Jones protein, CD0000077000; prealbumin, CD0000091000; eGFP, CD0004251000; MAGI-1PDZ1, CD0000596000; UTPase, CD0003897000; ecotin, CD0003896000; trypsin, CD0000096000; and α-lactalbumin, CD0000072000. Amelogenin, Sic1, β−B1 C-terminus_1-94_, pokeweed lectin and saporin have consecutive records CD0006410000 to CD0006414000 and are identified by the keyword “IDPtest”. The spectra of proteins present in the SP175 dataset are already available in the PCDDB, and are identified by the keyword “SP175”. Twelve existing SP175 entries have been updated for this project; these are identified by a “1” in the 10th position of the PCDDBID. The PCDDB accession codes for each protein, and their secondary structures that were used in creating the IDP reference dataset are listed in Supplementary Tables S1 (top), S1 (bottom) and S2.

## Code availability

The DichroIDP app is freely available for download at: https://dichroidp.cryst.bbk.ac.uk and from GitHub at https://github.com/pcddb/DichroIDPs.

## References

[1] Uversky VN, Gillespie JR, Fink AL (2000). Why are “natively unfolded” proteins unstructured under physiologic conditions?. Proteins Struct. Funct. Bioinf..

[2] van der Lee R (2014). Classification of intrinsically disordered regions and proteins. Chem. Rev..

[3] Dyson HJ, Wright PE (2005). Intrinsically unstructured proteins and their functions. Nat. Rev. Mol. Cell Biol..

[4] Haynes C (2006). Intrinsic disorder is a common feature of hub proteins from four eukaryotic interactomes. PLoS Comp. Biol..

[5] Miles AJ, Wallace BA (2006). Synchrotron radiation circular dichroism spectroscopy of proteins and applications in structural and functional genomics. Chem. Soc. Rev..

[6] Miles AJ, Janes RW, Wallace BA (2021). Tools and methods for circular dichroism spectroscopy of proteins: a tutorial review. Chem. Soc. Rev..

[7] Whitmore L, Wallace BA (2008). Protein secondary structure analyses from circular dichroism spectroscopy: Methods and reference databases. Biopolymers.

[8] Lees JG, Miles AJ, Wien F, Wallace BA (2006). A reference database for circular dichroism spectroscopy covering fold and secondary structure space. Bioinformatics.

[9] Abdul-Gader A, Miles AJ, Wallace BA (2011). A reference dataset for the analyses of membrane protein secondary structures and transmembrane residues using circular dichroism spectroscopy. Bioinformatics.

[10] Sreerama N, Venyaminov SY, Woody RW (2000). Estimation of protein secondary structure from CD spectra: Inclusion of denatured proteins with native proteins in the analysis. Anal. Biochem..

[11] Miles AJ, Ramalli SG, Wallace BA (2021). DichroWeb, a website for calculating protein secondary structure from circular dichroism spectroscopic data. Protein Sci..

[12] Micsonai A (2018). BeStSel: A web server for accurate protein secondary structure prediction and fold recognition from the circular dichroism spectra. Nucleic Acids Res..

[13] Nagy G, Igaev M, Jones NC, Hoffmann SV, Grubmüller H (2019). SESCA: Predicting circular dichroism spectra from protein molecular structures. J. Chem. Theory Comput..

[14] Sreerema N, Woody RW (1993). A self-consistent method for the analysis of protein secondary structure from circular dichroism. Anal. Biochem..

[15] Provencher SW, Glöckner J (1981). Estimation of globular protein secondary structure from circular dichroism. Biochemistry.

[16] Compton LA, Johnson WC (1986). Analysis of protein circular dichroism spectra for secondary structure using a simple matrix multiplication. Anal. Biochem..

[17] Micsonai A (2015). Accurate secondary structure prediction and fold recognition for circular dichroism spectroscopy. Proc. Nat. Acad. Sci..

[18] Hanson J, Paliwal K, Litfin T, Yang Y, Zhou Y (2019). Improving prediction of protein secondary structure, backbone angles, solvent accessibility and contact numbers by using predicted contact maps and an ensemble of recurrent and residual convolutional neural networks. Bioinformatics.

[19] Klausen MS (2019). NetSurfP-2.0: Improved prediction of protein structural features by integrated deep learning. Proteins Struct. Funct. Bioinf..

[20] Källberg M (2012). Template-based protein structure modelling using the RaptorX web server. Nat. Protoc..

[21] Jumper J (2021). Highly accurate protein structure prediction with AlphaFold. Nature.

[22] Burley SK (2021). RCSB Protein Data Bank: powerful new tools for exploring 3D structures of biological macromolecules for basic and applied research and education in fundamental biology, biomedicine, biotechnology, bioengineering and energy sciences. Nucleic Acids Res..

[23] Kabsch W, Sander C (1983). Dictionary of Protein Secondary Structure: Pattern recognition of hydrogen-bonded geometrical features. Biopolymers.

[24] Senior AW (2020). Improved protein structure prediction using potentials from deep learning. Nature.

[25] David A, Islam S, Tankhilevich E, Sternberg MJE (2022). The AlphaFold Database of Protein Structures: A Biologist’s Guide. J. Mol. Biol..

[26] Ruff KM, Pappu RV (2021). AlphaFold and implications for intrinsically disordered proteins. J. Mol. Biol..

[27] Wilson CJ, Choy W-Y, Karttunen M (2022). AlphaFold2: A role for disordered protein/region prediction?. Int. J. Mol. Sci..

[28] Ramalli SG, Miles AJ, Janes RW, Wallace BA (2022). The PCDDB (Protein Circular Dichroism Data Bank): A bioinformatics resource for protein characterisations and methods development. J. Mol. Biol..

[29] Louis-Jeune C, Andrade-Navarro MA, Perez-Iratxeta C (2012). Prediction of protein secondary structure from circular dichroism using theoretically derived spectra. Proteins.

[30] Panethymitaki, C. Kinetoplastid myristoyl CoA: protein N-myristoyltransferase and two substrates, the *Leishmania* vaccine antigen candidates, HASPA and HASPB. *PhD Thesis*, Imperial College London. (2005).

[31] Micsonai A (2022). Disordered–ordered protein binary classification by circular dichroism spectroscopy. Front. Mol. Biosci..

[32] Bulheller BM, Hirst JD (2009). DichroCalc – circular and linear dichroism online. Bioinformatics.

[33] Lopes JLS, Orcia D, Araujo APU, DeMarco R, Wallace BA (2013). Folding factors and partners for the intrinsically disordered protein micro-exon gene 14 (MEG-14). Biophys. J..

[34] Richards, M. W. Structural studies of a Ca++ channel beta subunit using biophysical methods. *PhD Thesis*, Birkbeck College, University of London (2004).

[35] Richards MW (2002). Synchrotron radiation circular dichroism and circular dichroism spectroscopic studies for the voltage-dependent calcium channel beta subunit. Biophys. J..

[36] Brocca S (2009). Order propensity of an intrinsically disordered protein, the cyclin-dependent-kinase inhibitor Sic1. Proteins.

[37] Lacruz RS (2011). Structural analysis of a repetitive protein sequence motif in strepsirrhine primate amelogenin. PLoS One..

[38] Rohatgi, A. WebPlotDigitizer at URL https://automeris.io/WebPlotDigitizer, Version: 4.5, (2021).

[39] The UniProt Consortium. UniProt: The universal protein knowledgebase in 2021. *Nucleic Acids Res*. **49**, D480–D489 (2021). 10.1093/nar/gkaa1100 PMC7778908 33237286

[40] Gasteiger E (2003). ExPASy: The proteomics server for in-depth protein knowledge and analysis. Nucleic Acids Res..

[41] Sutherland, J. Circular Dichroism and the Conformational Analysis of Biomolecules. (Plenum Press, 1996). 616–618.

[42] Anthis NJ, Clore GM (2013). Sequence-specific determination of protein and peptide concentrations by absorbance at 205 nm. Protein Sci..

[43] Miles AJ, Wien F, Lees JG, Wallace BA (2005). Calibration and standardisation of synchrotron radiation and conventional circular dichroism spectrometers. Part 2: Factors affecting magnitude and wavelength. Spectroscopy.

[44] Miles AJ, Wallace BA (2018). CDtoolX, a downloadable software package for processing and analyses of circular dichroism spectroscopic data. Protein Sci..

[45] Miles AJ (2003). Calibration and standardisation of synchrotron radiation circular dichroism and conventional circular dichroism spectrophotometers. Spectroscopy.

[46] Klose DP, Wallace BA, Janes RW (2010). 2Struc: The secondary structure server. Bioinformatics.

[47] The Qt Company. https://www.qt.io/.

[48] Bochkanov, S. A. ALGLIB. http://www.alglib.net.

[49] MATLAB [7.0]. MathWorks, 2005.

