# Supplementary material for: DichroIDP: a method for analyses of intrinsically disordered proteins using circular dichroism spectroscopy
Source: Commun Biol. 2023 Aug 8;6:823. doi: 10.1038/s42003-023-05178-2 (PMC10409736; doi:10.1038/s42003-023-05178-2)

Supplementary Information for DichroIDP: A Novel Method for Analyses for Intrinsically Disordered Proteins Using Circular Dichroism Spectroscopy
(Miles, Drew, and Wallace)

Supplementary Tables and Supplementary Figure S1

Supplementary Table S1 (Top).
Secondary structures assigned for IDP proteins included to create the IDP175 reference data set (RDS). The secondary structures were predicted by AlphaFold2[21] and described in the Methods section with DSSP[23]-defined values for helix (H), sheet (E), turn (T), and disordered (D) structures. The corresponding spectroscopic and metadata have been deposited in the Protein Circular Dichroism Data Bank[28] (PCDDB) (entry IDs are given in column 7).

Protein	Uniprot ID	Secondary structures (%) derived from AlphaFold2 structure				PCDDB ID
		H	E	T	D	
MEG-14	M1GUG5	0	0	0	100	CD0004064000
HASPA	P90552	0	0	3	97	CD0006406000
HASPB	Q25326	0	0	3	97	CD0006407000
$TARP_{174-222}$	Q6GX35	7	0	16	77	CD0006408000
Beta-casein	P02666	28	0	7	64	CD0006409000

Supplementary Table S1 (Bottom).

Secondary Structures of the Test Proteins. All of the spectroscopic and metadata for these proteins have been deposited in the Protein Circular Dichroism Data Bank. [H= helix, E= sheet, T=turn, D=disordered]

**Protein Type**	**Uniprot ID**	**Secondary Structures (%) from AlphaFold2/Crystal Structure**				**PCDDB ID**
Protein Name		**H**	**E**	**T**	**D**	
**Disordered**						
Osteopontin	P10451	9	0	1	91	CD000366700
Amelogenin	A0A6P5P1L3	15	0	7	78	CD000641000
Sic1Inhibitor	P38634	13	4	10	74	CD000641100
BB1 Cterm$_{1\text{-}94}$	-	23	0	6	72	CD000641200
**Mostly β1**						
b2- Microglobulin	P61769	0	47	21	32	CD0003894000
Prealbumin	P02766	6	45	24	26	CD0000091000
Bence Jones	P01699	5	47	23	25	CD0000077000
eGFP pH7.4	C5MKY7	22	44	20	15	CD0004251000
**Mostly β2**						
MAGI-1 PDZ1	Q96QZ7	14	25	22	38	CD0005960000
UTPase	P33316	6	35	23	37	CD0003897000
Ecotin	P23827	8	45	12	36	CD0003896000
Trypsin	P00760	10	29	27	35	CD0000096000
**Mostly α/β**						
Pokeweed	Q9AVB0	17	7	41	36	CD0006413000
Saporin	P20656	34	17	19	29	CD0006414000
**Mostly α**						
α-Lactalbumin	P00709	40	6	23	32	CD0000072000

.

Supplementary Table S2. Secondary Structure Assignments and PCDDB IDs for the SP175 Database Proteins. The secondary structures are as defined in the Methods Section using DSSP[23] values for Helix (H), Sheet (E), Turn (T), and Disordered (D). These values were calculated from their crystallographic entries in the PDB[22] (second column). Their spectral data are available in the PCDDB[28]. (Note that for some entries in the PCDDB the PCDDBid contains a "1" in the 10th position of the name, indicating a revised spectra). Five proteins from the original SP175[8] dataset are not included (see text). These are the proteins containing highly-twisted, right-handed antiparallel beta sheets that give rise to β2-like spectra: alpha-chymotrypsin, alpha-chymotrypsinogen, elastase, and soybean trypsin inhibitor. Ferredoxin was also omitted due to its anomalous β2-like spectrum.

Protein	PDB ID	Secondary structures (%)				PCDDB ID
		H	E	T	D	
Aldolase	1ado	46	14	16	24	CD0000001000
Alkaline phosphatase	1ed9	31	19	25	25	CD0000002100
Alpha amylase	1vjs	29	21	24	27	CD0000003000
Alpha bungarotoxin	1hc9	5	37	27	31	CD0000004000
Aprotinin	5pti	21	24	22	33	CD0000007000
Avidin	1rav	7	46	16	32	CD0000008100
Beta amylase	1fa2	38	11	22	29	CD0000009000
Beta galactosidase	1dp0	14	38	22	26	CD0000010000
Beta lactoglobulin	3blg	14	38	28	19	CD0000011000
Beta-B2 crystallin	2bb2	7	43	17	32	CD0000022000
Gamma-B crystallin	4gcr	9	46	20	25	CD0000023000
Gamma-D crystallin	1elp	9	43	25	23	CD0000024000
Gamma-E-crystallin	1m8u	6	43	24	26	CD0000025000
c-Phycocyanin	1ha7	78	0	12	10	CD0000012000
Calmodulin	1lin	57	5	19	19	CD0000013000
Carbonic anhydrase I	1hcb	18	29	26	27	CD0000014000
Carbonic anhydrase II	1v9e	16	29	28	28	CD0000015000
Carboxypeptidase A1	5cpa	38	16	25	21	CD0000016000
Catalase	1dgf	34	17	25	25	CD0000017000
Ceruloplasmin	1kcw	12	33	25	30	CD0000018000
Citrate synthase	1cts	60	1	17	22	CD0000019100
Concanavalin A	1nls	4	46	25	25	CD0000020000
Cytochrome C	1hrc	41	0	25	34	CD0000021000
3-dehydroquinate dehydratase	1qfe	46	19	18	18	CD0000028000
3-dehydroquinate dehydratase	2dhq	44	17	16	23	CD0000029000
Deoxyribonuclease-1	3dni	29	26	18	26	CD0000030000
Gamma-s-crystallin Ct	1ha4	10	45	22	23	CD0000026000
Glucose oxidase	1cf3	34	19	21	25	CD0000033000
Glutamate dehydrogenase I	3mw9	48	12	21	18	CD0000034000
Glycogen phosphorylase-b	1gpb	51	15	17	17	CD0000035100
Haloalkane dehalogenase	1bn6	46	18	17	18	CD0000036000
Hemoglobin	1hda	76	0	12	12	CD0000037100
Human serum albumin	1n5u	72	0	15	13	CD0000038100
Gamma-D crystallin (Human)	1hk0	8	49	21	22	CD0000027100

Immunoglobulin G	1igt	7	44	19	30	CD0000039100
Insulin	1trz	67	0	5	29	CD0000040000
Jacalin	1ku8	0	66	14	20	CD0000041000
Lactoferrin	1blf	33	18	31	18	CD0000042000
Lectin (lentil)	1les	3	44	25	27	CD0000043000
Leptin	1ax8	61	0	8	31	CD0000044000
Lysozyme	193l	40	6	33	20	CD0000045000
Monellin	1mol	18	53	13	16	CD0000046000
Myoglobin	1ymb	75	0	14	11	CD0000047000
Myoglobin	1a6m	78	0	11	11	CD0000048000
NmrA	1k6j	37	16	20	27	CD0000049000
Ovalbumin	1ova	29	29	14	28	CD0000050000
Ovotransferrin	1dot	30	17	32	21	CD0000051000
Papain	1ppn	26	18	24	33	CD0000052000
Lectin (pea)	1ofs	3	43	25	30	CD0000053000
Pectate lyase C	1air	13	32	26	29	CD0000054000
Pepsinogen	2psg	15	36	19	30	CD0000055000
Peroxidase C1	7atj	50	2	28	20	CD0000056000
Phosphoglucomutase 1	3pmg	36	25	21	18	CD0000057000
Phosphoglycerate kinase	3pgk	35	11	27	28	CD0000058000
Phospholipase A2	1une	50	7	22	22	CD0000059000
PNMT	1hnn	34	22	19	25	CD0000060000
Pyruvate Kinase	1a49	39	18	22	21	CD0000061100
Rhodanese	1rhs	33	13	25	30	CD0000062000
Ribonuclease, pancreatic	3rn3	21	33	25	21	CD0000063100
Rubredoxin	1r0i	17	15	28	41	CD0000064000
Streptavidin	1stp	8	55	17	20	CD0000066000
Subtilisin Carlsberg	1scd	29	17	27	27	CD0000067100
Superoxide Dismutase	1cbj	4	39	30	27	CD0000068000
Thaumatin I	1thw	11	36	27	27	CD0000069000
Triose Phosphate Isomerase	7tim	45	16	17	22	CD0000070000
Ubiquitin	1ubi	25	32	18	25	CD0000071000

<u>Supplementary Table S3.</u> **Cross validation results for the disordered secondary structure fraction**, as produced using different methods for calculating the secondary structure fraction of the IDPs in the IDP175 RDS. The statistical parameters, r, δ, ζ, are described in the main text.

Method	r	δ	ζ
**AlphaFold2**	0.9322	0.0649	2.6987
**NetSurfP**	0.9253	0.0712	2.5881
**RaptorX**	0.9129	0.0715	2.4265
**Spot1D**	0.8870	0.0795	2.0897

<u>Supplementary Table S4.</u> **Secondary structures (%) of proteins that were not included in the data base, whose spectra are shown in Figure S1.** The secondary structures are as predicted by Alphafold2 (see text for explanation).

		**Alphafold2-defined Secondary Structure (%)**		
**Protein**	**H**	**E**	**T**	**D**
SHERP	90	0	2	8
a-synuclein	45	0	20	35
b-synuclein	45	0	20	35
TARP $_{726\text{-}825}$	26	0	16	58

<u>Supplementary Table S5.</u> Calculated secondary structures for the test proteins using DichroIDP with the new IDP175 reference data sets, and (from left to right) the existing SP175/SP175t[8], CDPro42[10] and SP175+[17] reference data sets. NRMSD is the "goodness-of-fit parameter" (see main text and reference 11). The designations of the secondary structure are H= helix, E= sheet, T=turn, D=disordered. The low-wavelength cutoffs of the data used are in indicated (in rows 3 and 4, and for of each entry as either 190 nm or the lowest wavelength in the spectrum (if lower than 190 nm). CDPro42 does not calculate results down to 175 nm.

	IDP175/190					SP175/SP175t[8]					CDPro42[10]					SP175+[17] (BeStSel-modified SP175)[12]				
**Osteopontin**	Mostly disordered																			
**Method/cutoff**	**H**	**E**	**T**	**D**	**NRMSD**	**H**	**E**	**T**	**D**	**NRMSD**	**H**	**E**	**T**	**D**	**NRMSD**	**H**	**E**	**T**	**D**	**NRMSD**
**DSSP/ AlphaFold2**	9	0	1	91		9	0	1	91		9	0	1	91		9	0	1	91	
**DSSP/PDB**	-	-	-	-		-	-	-	-		-	-	-	-		-	-	-	-	
**DichroIDP 190**	7	1	8	84	0.034	12	25	22	40	0.011	6	6	5	82	0.011	12	25	23	40	0.050
**DichroIDP 175**	7	0	10	81	0.083	11	29	19	41	0.011	-	-	-	-		11	29	19	41	0.011
**Sic1**	Mostly disordered																			
**Method/cutoff**	**H**	**E**	**T**	**D**	**NRMSD**	**H**	**E**	**T**	**D**	**NRMSD**	**H**	**E**	**T**	**D**	**NRMSD**	**H**	**E**	**T**	**D**	**NRMSD**
**DSSP/ AlphaFold2**	13	4	10	74		13	4	10	74		13	4	10	74		13	4	10	74	
**DSSP/PDB**	-	-	-	-		-	-	-	-		-	-	-	-		-	-	-	-	
**DichroIDP 190**	12	9	13	65	0.041	14	32	24	29	0.000	4	17	13	62	0.029	14	32	24	29	0.000
**Amelogenin**	Mostly disordered																			
**Method/cutoff**	**H**	**E**	**T**	**D**	**NRMSD**	**H**	**E**	**T**	**D**	**NRMSD**	**H**	**E**	**T**	**D**	**NRMSD**	**H**	**E**	**T**	**D**	**NRMSD**
**DSSP/ AlphaFold2**	15	0	7	78		15	0	7	78		15	0	7	78		15	0	7	78	
**DSSP/PDB**	-	-	-	-		-	-	-	-		-	-	-	-		-	-	-	-	
**DichroIDP 190**	16	5	15	64	0.033	16	22	26	37	0.033	9	27	20	43	0.031	17	18	25	38	0.032
**BB1 1-94**	Mostly disordered																			
**Method/cutoff**	**H**	**E**	**T**	**D**	**NRMSD**	**H**	**E**	**T**	**D**	**NRMSD**	**H**	**E**	**T**	**D**	**NRMSD**	**H**	**E**	**T**	**D**	**NRMSD**
**DSSP/ AlphaFold2**	23	0	6	72		23	0	6	72		23	0	6	72		23	0	6	72	
**DSSP/PDB**	--	-	-	-		-	-	-	-		-	-	-	-		-	-	-	-	
**DichroIDP 190**	8	-1	9	85	0.020	13	21	25	42	0.084	9	14	13	64	0.009	16	22	26	37	0.014
**DichroIDP 175**	8	6	11	74	0.015	13	19	26	43	0.016	-	-	-	-		10	23	25	42	0.020

β-2 microglobulin Mostly β1

Method/cutoff	H	E	T	D	NRMSD	H	E	T	D	NRMSD	H	E	T	D	NRMSD	H	E	T	D	NRMSD
**DSSP/ AlphaFold2**	0	47	21	32		0	47	21	32		0	47	21	32		0	47	21	32	
**DSSP / PDB 2YXF**	3	48	17	32		3	48	17	32		3	48	17	32		3	48	17	32	
**DichroIDP 190**	7	45	19	29	0.071	7	45	19	29	0.071	0	48	27	26	0.026	3	48	17	31	0.050
**DichroIDP 175**	6	47	19	29	0.047	6	47	18	29	0.047	-	-	-	-		3	49	17	30	0.029

Prealbumin Mostly β1

Method/cutoff	H	E	T	D	NRMSD	H	E	T	D	NRMSD	H	E	T	D	NRMSD	H	E	T	D	NRMSD
**DSSP/ AlphaFold2**	6	45	24	26		6	45	24	26		6	45	24	26		6	45	24	26	
**DSSP / PDB 2PAB**	6	47	20	28		6	47	20	28		6	47	20	28		6	47	20	28	
**DichroIDP 190**	12	37	23	26	0.067	12	37	23	26	0.067	8	47	20	25	0.043	12	35	25	25	0.070
**DichroIDP 180**	14	37	22	28	0.053	14	37	22	28	0.056	-	-	-	-		14	36	22	28	0.001

Bence Jones protein Mostly β1

Method/cutoff	H	E	T	D	NRMSD	H	E	T	D	NRMSD	H	E	T	D	NRMSD	H	E	T	D	NRMSD
**DSSP/ AlphaFold2**	5	47	23	25		5	47	23	25		5	47	23	25		5	47	23	25	
**DSSP / PDB 2RHE**	3	43	27	27		3	43	27	27		3	43	27	27		3	43	27	27	
**DichroIDP 190**	7	44	19	30	0.000	7	44	19	30	0.000	4	42	23	30	0.028	7	44	19	30	0.000
**DichroIDP 175**	15	36	19	30	0.013	15	36	19	30	0.013	-	-	-	-		15	36	19	30	0.013

eGFP Mostly β1

Method/cutoff	H	E	T	D	NRMSD	H	E	T	D	NRMSD	H	E	T	D	NRMSD	H	E	T	D	NRMSD
**DSSP/ AlphaFold2**	22	44	20	15		22	44	20	15		22	44	20	15		22	44	20	15	
**DSSP/PDB**	-	-	-	-		-	-	-	-		-	-	-	-		-	-	-	-	
**DichroIDP 190**	12	40	24	24	0.106	12	40	24	25	0.106	11	42	24	24	0.017	15	35	24	25	0.039
**DichroIDP 182**	14	37	24	26	0.125	14	37	24	25	0.125	-	-	-	-		15	36	24	26	0.044

MAGI 1PDZ1 Mostly β2

Method/cutoff	H	E	T	D	NRMSD	H	E	T	D	NRMSD	H	E	T	D	NRMSD	H	E	T	D	NRMSD
**DSSP/ AlphaFold2**	14	25	22	38		14	25	22	38		14	25	22	38		14	25	22	38	
**DSSP / PDB 2KPK**	12	32	31	26		12	32	31	26		12	32	31	26		12	32	31	26	

**DichroIDP 190**	20	15	15	49	0.090	19	26	21	35	0.089	22	21	21	36	0.049	18	26	21	35	0.089

UTPase Mostly β2

**Method/cutoff**	**H**	**E**	**T**	**D**	**NRMSD**	**H**	**E**	**T**	**D**	**NRMSD**	**H**	**E**	**T**	**D**	**NRMSD**	**H**	**E**	**T**	**D**	**NRMSD**
**DSSP/ AlphaFold2**	6	35	23	37		6	35	23	37		6	35	23	37		6	35	23	37	
**DSSP / PDB 1Q5U**	5	39	18	38		5	39	18	38		5	39	18	38		5	39	18	38	
**DichroIDP 190**	15	23	13	49	0.029	12	33	22	32	0.027	15	24	19	43	0.023	11	34	22	32	0.055
**DichroIDP 175**	9	33	26	30	0.004	12	33	24	32	0.003	-	-	-	-		11	34	24	33	0.055

Ecotin Mostly β2

**Method/cutoff**	**H**	**E**	**T**	**D**	**NRMSD**	**H**	**E**	**T**	**D**	**NRMSD**	**H**	**E**	**T**	**D**	**NRMSD**	**H**	**E**	**T**	**D**	**NRMSD**
**DSSP/ AlphaFold2**	8	45	12	36		8	45	12	36		8	45	12	36		8	45	12	36	
**DSSP / PDB 1ECZ**	2	40	18	40		2	40	18	40		2	40	18	40		2	40	18	40	
**DichroIDP 190**	21	5	12	62	0.003	15	27	24	35	0.083	14	31	23	31	0.017	14	30	24	33	0.045
**DichroIDP 178**	28	0	7	64	0.002	12	31	24	34	0.007	-	-	-	-		12	11	24	34	0.007

Trypsin Mostly β2

**Method/cutoff**	**H**	**E**	**T**	**D**	**NRMSD**	**H**	**E**	**T**	**D**	**NRMSD**	**H**	**E**	**T**	**D**	**NRMSD**	**H**	**E**	**T**	**D**	**NRMSD**
**DSSP/ AlphaFold2**	10	29	27	35		10	29	27	35		10	29	27	35		10	29	27	35	
**DSSP / PDB 1AUJ**	10	29	26	35		10	29	26	35		10	29	26	35		10	29	26	35	
**DichroIDP 190**	9	16	15	59	0.030	13	29	23	36	0.073	7	25	18	51	0.099	13	29	23	36	0.105
**DichroIDP 175**	0	0	0	1	0.004	10	30	19	41	0.000	-	-	-	-		10	30	19	41	0.000

Pokeweed lectin Mostly α/β

**Method/cutoff**	**H**	**E**	**T**	**D**	**NRMSD**	**H**	**E**	**T**	**D**	**NRMSD**	**H**	**E**	**T**	**D**	**NRMSD**	**H**	**E**	**T**	**D**	**NRMSD**
**DSSP/ AlphaFold2**	17	7	41	36		17	7	41	36		17	7	41	36		17	7	41	36	
**DSSP/PDB**	-	-	-	-		-	-	-	-		-	-	-	-		-	-	-	-	
**DichroIDP 190**	6	26	23	45	0.102	9	34	26	32	0.091	4	39	30	26	0.000	9	32	27	32	0.032
**DichroIDP 180**	1	39	30	32	0.008	14	32	24	29	0.000	-	-	-	-		14	32	24	29	0.000

Saporin Mostly α/β

**Method/cutoff**	**H**	**E**	**T**	**D**	**NRMSD**	**H**	**E**	**T**	**D**	**NRMSD**	**H**	**E**	**T**	**D**	**NRMSD**	**H**	**E**	**T**	**D**	**NRMSD**
**DSSP/ AlphaFold2**	33	17	19	29		33	17	19	29		33	17	19	29		33	17	19	29	

**DSSP / PDB 1QI7**	37	20	20	24		37	20	20	34		37	20	20	24		37	20	20	24	
**DichroIDP 190**	29	24	24	23	0.079	30	23	24	22	0.079	28	23	23	26	0.033	26	26	23	23	0.069
**DichroIDP 175**	33	15	28	23	0.050	33	15	28	23	0.050	-	-	-	-		32	17	27	23	0.050
**α-lactalbumin**	Mostly α																			
**Method/cutoff**	**H**	**E**	**T**	**D**	**NRMSD**	**H**	**E**	**T**	**D**	**NRMSD**	**H**	**E**	**T**	**D**	**NRMSD**	**H**	**E**	**T**	**D**	**NRMSD**
**DSSP/ AlphaFold2**	40	6	23	32		40	6	23	32		40	6	23	32		40	6	23	32	
**DSSP/PDB 1HML**	43	7	29	22		43	7	29	22		43	7	29	22		43	7	29	22	
**DichroIDP 190**	39	7	27	25	0.060	39	8	27	25	0.054	40	5	20	34	0.064	39	8	28	25	0.054
**DichroIDP 175**	37	13	25	24	0.021	37	13	26	23	0.021	-	-	-	-		39	11	27	22	0.026

Notes: An NRMSD of 0.000 for DichroIDP can arise when the value is very small (>0.001), usually resulting from inaccurate concentration or pathlength values used during data collection and could be rectified by scaling the spectrum using the scaling function in DichroIDP.

Supplementary Table S6. Calculated secondary structures for the test proteins using BeStSel[12] (left) and K2D3[29] (right) methods. The designations of the secondary structure types by each method are as described in references 12 [where "Turn" and "Disorder" are defined differently by the BeStSel method from those parameters in Supplementary Tables S5 and S7] and 29, respectively, where H= helix, E= sheet, T=turn, D=disordered, O=other.

**BeStSel**						**K2D3**			
**Osteopontin**	Mostly disordered								
**Method/cutoff**	**H**	**E**	**T**	**D**	**NRMSD**		**H**	**E**	**O**
**DSSP/ AlphaFold2**	9	0	1	91		**DSSP/ AlphaFold2**	9	0	92
**DSSP/PDB**	-	-	-	-		**DSSP/PDB**	-	-	-
**BeStSel**	4	27	20	49	0.042	**K2D3**	4	37	59
**Sic1**	Mostly disordered								
**Method/cutoff**	**H**	**E**	**T**	**D**	**NRMSD**		**H**	**E**	**O**
**DSSP/ AlphaFold2**	13	4	4	79		**DSSP/ AlphaFold2**	13	4	83
**DSSP/PDB**	-	-	-	-		**DSSP/PDB**	-	-	-
**BeStSel**	6	27	19	48	0.024	**K2D3**	4	37	59
**Amelogenin**	Mostly disordered								
**Method/cutoff**	**H**	**E**	**T**	**D**	**NRMSD**		**H**	**E**	**O**
**DSSP/ AlphaFold2**	15	0	4	81		**DSSP/ AlphaFold2**	15	0	85
**DSSP/PDB**	-	-	-	-		**DSSP/PDB**	-	-	-
**BeStSel**	14	23	20	49	0.017	**K2D3**	5	31	64
**BB1 1-94**	Mostly disordered								
**Method/cutoff**	**H**	**E**	**T**	**D**	**NRMSD**		**H**	**E**	**O**
**DSSP/ AlphaFold2**	23	0	2	75		**DSSP/ AlphaFold2**	23	0	77
**DSSP/PDB**	--	-	-	-		**DSSP/PDB**	-	-	-
**BeStSel**	4	26	19	48	0.024	**K2D3**	4	33	63
**β-2 microglobulin**	Mostly β1								
**Method/cutoff**	**H**	**E**	**T**	**D**	**NRMSD**		**H**	**E**	**O**
**DSSP/ AlphaFold2**	0	47	10	43		**DSSP/ AlphaFold2**	0	47	53
**DSSP / PDB 2YXF**	3	48	9	41		**DSSP/PDB 2YXF**	3	48	49
**BeStSel**	0	48	10	41	0.023	**K2D3**	1	43	56
**Prealbumin**	Mostly β1								
**Method/cutoff**	**H**	**E**	**T**	**D**	**NRMSD**		**H**	**E**	**O**
**DSSP/ AlphaFold2**	6	45	16	34		**DSSP/ AlphaFold2**	6	45	49
**DSSP / PDB 2PAB**	6	46	9	36		**DSSP/PDB 2PAB**	6	46	48
**BeStSel**	8	48	10	44	0.010	**K2D3**	4	40	56
**Bence Jones protein**	Mostly β1								
**Method/cutoff**	**H**	**E**	**T**	**D**	**NRMSD**		**H**	**E**	**O**
**DSSP/ AlphaFold2**	3	47	19	31		**DSSP/ AlphaFold2**	3	47	50
**DSSP / PDB 2RHE**	3	43	20	34		**DSSP/PDB 2RHE**	3	43	54
**BeStSel**	5	42	12	41	0.024	**K2D3**	2	42	56

eGFP	Mostly β1								
**Method/cutoff**	**H**	**E**	**T**	**D**	**NRMSD**		**H**	**E**	**O**
**DSSP/ AlphaFold2**	22	44	12	22		**DSSP/ AlphaFold2**	22	44	34
**DSSP/PDB**	-	-	-	-		**DSSP/PDB**	-	-	-
**BeStSel**	13	33	11	43	0.084	**K2D3**	6	41	53

MAGI 1PDZ1	Mostly β2								
**Method**	**H**	**E**	**T**	**D**	**NRMSD**		**H**	**E**	**O**
**DSSP/ AlphaFold2**	14	25	14	47		**DSSP/ AlphaFold2**	14	25	61
**DSSP /PDB 2KPK**	12	32	15	42		**DSSP/PDB 2KPK**	12	32	56
**BeStSel**	9	21	14	56	0.024	**K2D3**	13	21	66

UTPase	Mostly β2								
**Method**	**H**	**E**	**T**	**D**	**NRMSD**		**H**	**E**	**O**
**DSSP/ AlphaFold2**	6	35	11	49		**DSSP/ AlphaFold2**	6	35	59
**DSSP /PDB 1Q5U**	5	39	10	47		**DSSP/PDB 1Q5U**	5	39	56
**BeStSel**	1	36	10	53	0.027	**K2D3**	8	29	63

Ecotin	Mostly β2								
**Method**	**H**	**E**	**T**	**D**	**NRMSD**		**H**	**E**	**O**
**DSSP/ AlphaFold2**	8	45	8	40		**DSSP/ AlphaFold2**	8	45	47
**DSSP / PDB 1ECZ**	2	40	9	49		**DSSP/PDB 1ECZ**	2	40	58
**BeStSel**	8	35	5	50	0.039	**K2D3**	7	26	67

trypsin	mostly β2								
**Method**	**H**	**E**	**T**	**D**	**NRMSD**		**H**	**E**	**O**
**DSSP/ AlphaFold2**	10	29	14	47		**DSSP/ AlphaFold2**	10	29	61
**DSSP /PDB 1AUJ**	10	29	13	48		**DSSP/PDB 1AUJ**	10	29	61
**BeStSel**	8	33	17	43	0.029	**K2D3**	4	36	60

Pokeweed lectin	mostly α/β								
**Method**	**H**	**E**	**T**	**D**	**NRMSD**		**H**	**E**	**O**
**DSSP/ AlphaFold2**	17	7	23	54		**DSSP/ AlphaFold2**	17	7	76
**DSSP/PDB**	-	-	-	-		**DSSP/PDB**	-	-	-
**BeStSel**	14	28	15	43	0.044	**K2D3**	3	38	59

Saporin	mostly α/β								
**Method**	**H**	**E**	**T**	**D**			**H**	**E**	**O**
**DSSP/ AlphaFold2**	33	17	11	39		**DSSP/ AlphaFold2**	33	17	50
**DSSP /PDB 1QI7**	37	20	12	32		**DSSP/PDB 1Q17**	37	20	43
**BeStSel**	34	8	20	38	0.027	**K2D3**	27	21	52

α-lactalbumin	mostly α								
**Method**	**H**	**E**	**T**	**D**	**NRMSD**		**H**	**E**	**O**
**DSSP/ AlphaFold2**	40	6	14	41		**NRMSD**	40	6	54
**DSSP/PDB 1HML**	43	7	19	32			43	7	50
**BeStSel**	33	11	16	41	0.016	**K2D3**	32	16	52

<u>Supplementary Table S7.</u> Calculated secondary structures for the disordered test proteins using the SESCA method[13]. The basis spectra were derived from the IDP175 dataset (left) and DSSP-F (right). The designations of the secondary structure types are the same as those used the DichroIDP application [where H= helix, E= sheet, T=turn, D=disordered] as described in the methods section. DSSP-F is a dataset which comes as part of the SESCA package and is based on SP175. Side chain corrections were implemented in SESCA.

**Osteopontin**	**IDP175**							**DSSP-F**			
**Method**	**H**	**E**	**T**	**D**	**NRMSD**		**H**	**E**	**T**	**D**	**NRMSD**
**DSSP/ AlphaFold2**	9	0	1	91		**DSSP/ AlphaFold2**	9	0	1	91	
**DSSP/PDB**	-	-	-	-		**DSSP/PDB**	-	-	-		
**SESCA**	9	0	0	90	0.069	**SESCA**	1	25	36	38	0.087

**Sic1**	**IDP175**							**DSSP-F**			
**Method**	**H**	**E**	**T**	**D**	**NRMSD**		**H**	**E**	**T**	**D**	**NRMSD**
**DSSP/ AlphaFold2**	13	4	10	74		**DSSP/ AlphaFold2**	13	4	10	74	
**DSSP/PDB**	-	-	-	-		**DSSP/PDB**	-	-	-		
**SESCA**	14	0	11	75	0.027	**SESCA**	7	12	17	64	0.040

**Amelogenin**	**IDP175**							**DSSP-F**			
**Method**	**H**	**E**	**T**	**D**	**NRMSD**		**H**	**E**	**T**	**D**	**NRMSD**
**DSSP/ AlphaFold2**	15	0	7	78		**DSSP/ AlphaFold2**	15	0	7	78	
**DSSP/PDB**	-	-	-	-		**DSSP/PDB**	-	-	-		
**SESCA**	19	2	4	75	0.066	**SESCA**	11	15	5	70	0.081

**BB1 1-94**	**IDP175**							**DSSP-F**			
**Method**	**H**	**E**	**T**	**D**	**NRMSD**		**H**	**E**	**T**	**D**	**NRMSD**
**DSSP/ AlphaFold2**	23	0	6	72		**DSSP/ AlphaFold2**	23	0	6	72	
**DSSP/PDB**	--	-	-	-		**DSSP/PDB**	-	-	-	-	
**SESCA**	11	0	3	86	0.102	**SESCA**	3	15	11	71	0.057

Supplementary Figure S1. Spectra not included in the IDP dataset. All are typical for disordered spectra. Purple: SHERP; Blue: Alpha synuclein; Red: Beta synuclein; Green: $TARP_{726\text{-}825}$, but various analysis methods do not identify them as disordered (see text).

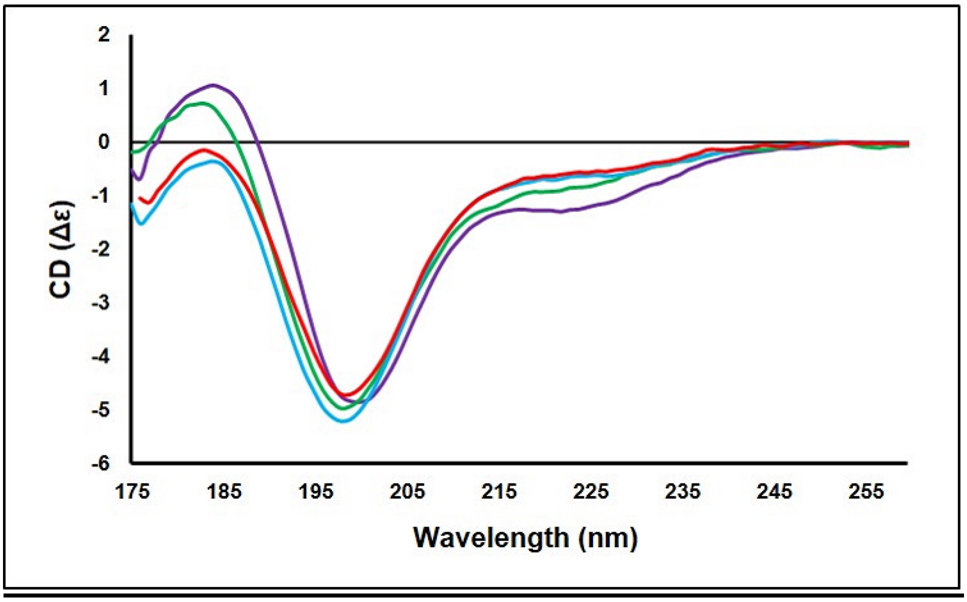

Supplement: Supplementary file 1 — Supplementary Information [file 42003_2023_5178_MOESM1_ESM.pdf]
